# Supplementary material for: Splice-Junction-Based Mapping of Alternative Isoforms in the Human Proteome
Source: Cell Rep. Author manuscript; Available in PMC 2020 Jan 15. (PMC6961840; doi:10.1016/j.celrep.2019.11.026)
Supplement: 2 [file NIHMS1546469-supplement-2.zip › DF1/PXD006675/Aorta/Aorta_1_TTN_APHVEFLRPLTDLQVR.pdf]

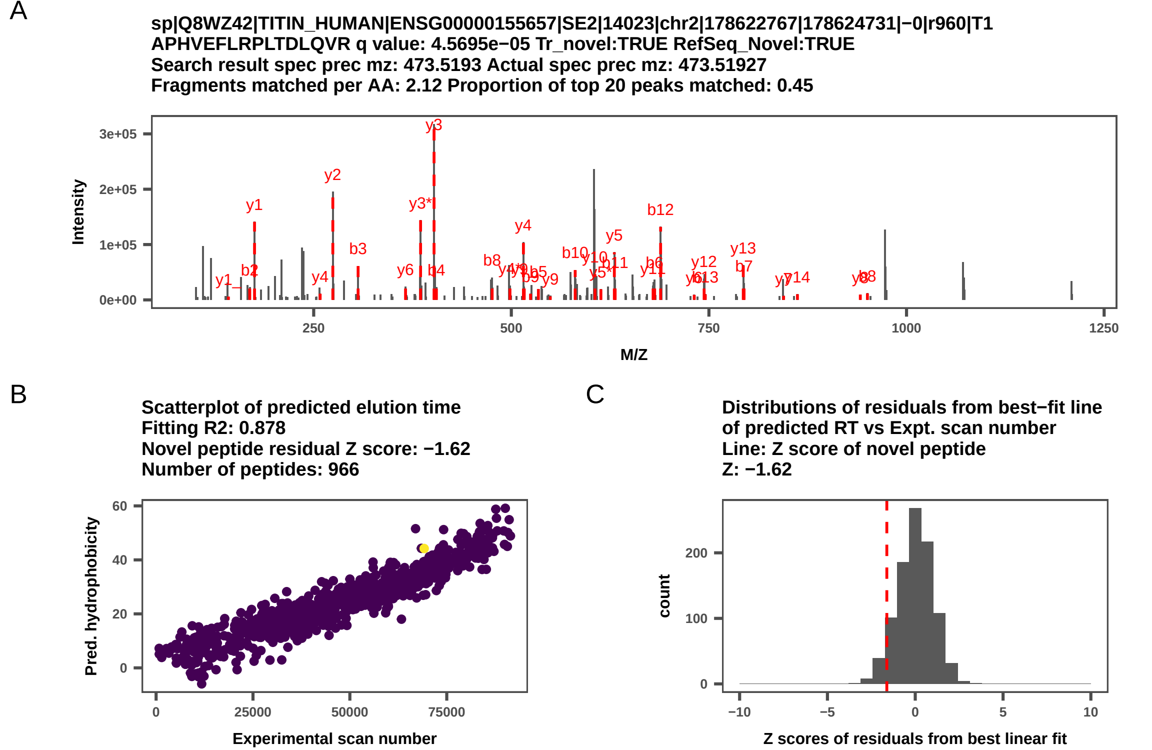
A
sp|Q8WZ42|TITIN_HUMAN|ENSG00000155657|SE2|14023|chr2|178622767|178624731|−0|r960|T1
APHVEFLRPLTDLQVR q value: 4.5695e−05 Tr_novel:TRUE RefSeq_Novel:TRUE
Search result spec prec mz: 473.5193 Actual spec prec mz: 473.51927
Fragments matched per AA: 2.12 Proportion of top 20 peaks matched: 0.45
Intensity
3e+05
2e+05
1e+05
0e+00
y3
y2
y1
y3*
b3
y4
b12
y5
y13
b7
b8
b10
b11
y12
y6
b4
y2
y4
y9
b13
y14
y8
250
500
750
1000
1250
M/Z
B
Scatterplot of predicted elution time
Fitting R2: 0.878
Novel peptide residual Z score: −1.62
Number of peptides: 966
Pred. hydrophobicity
60
40
20
0
0
25000
50000
75000
Experimental scan number
C
Distributions of residuals from best−fit line
of predicted RT vs Expt. scan number
Line: Z score of novel peptide
Z: −1.62
count
200
100
0
−10
−5
0
5
10
Z scores of residuals from best linear fit
